# Deep Learning-Based Intelligent Apple Variety Classification System and Model Interpretability Analysis

**DOI:** 10.3390/foods12040885

**Published:** 2023-02-19

**Authors:** Fanqianhui Yu, Tao Lu, Changhu Xue

**Affiliations:** 1 Haide College, Ocean University of China, Qingdao 266100, China; 2 Department of Computer Science and Technology, Ocean University of China, Qingdao 266100, China; 3 College of Food Science and Engineering, Ocean University of China, Qingdao 266003, China; 4 School of Mechanical and Automotive Engineering, Qingdao University of Technology, Qingdao 266520, China; 5 Laboratory of Marine Drugs and Biological Products, Pilot National Laboratory for Marine Science and Technology (Qingdao), Qingdao 266237, China

**Keywords:** apple varieties, Convolutional Neural Network, transfer learning, visualization methods, model interpretability

## Abstract

In this study, series networks (AlexNet and VGG-19) and directed acyclic graph (DAG) networks (ResNet-18, ResNet-50, and ResNet-101) with transfer learning were employed to identify and classify 13 classes of apples from 7439 images. Two training datasets, model evaluation metrics, and three visualization methods were used to objectively assess, compare, and interpret five Convolutional Neural Network (CNN)-based models. The results show that the dataset configuration had a significant impact on the classification results, as all models achieved over 96.1% accuracy on dataset A (training-to-testing = 2.4:1.0) compared to 89.4–93.9% accuracy on dataset B (training-to-testing = 1.0:3.7). VGG-19 achieved the highest accuracy of 100.0% on dataset A and 93.9% on dataset B. Moreover, for networks of the same framework, the model size, accuracy, and training and testing times increased as the model depth (number of layers) increased. Furthermore, feature visualization, strongest activations, and local interpretable model-agnostic explanations techniques were used to show the understanding of apple images by different trained models, as well as to reveal how and why the models make classification decisions. These results improve the interpretability and credibility of CNN-based models, which provides guidance for future applications of deep learning methods in agriculture.

## 1. Introduction

Fresh apples are one of the main fruits consumed worldwide due to their nutritional value and health benefits, with more than 7500 varieties available, the most popular ones including Granny Smith, Gala, Fuji, Red Delicious, Golden Delicious, and Braeburn [1,2]. Typically, several varieties of apples are grown simultaneously in an apple orchard, so it is easy to mix different varieties of apples with similar appearances during the harvesting and marketing process [3]. Although different varieties of apples are similar in external appearance, they have different intrinsic qualities in terms of taste and nutritional value, which is why people spend a lot of time sorting, packing, and labeling apples before selling them [4,5]. In addition, the identification and classification of apples is a necessary task, as it facilitates agricultural and industrial applications, such as automated apple grading systems, nutritional predictions, or meeting consumers’ dietary needs for specific apple varieties [6]. Traditional methods for apple variety recognition rely mainly on manual labor, which is subjective, slow, and unable to meet large-scale applications, while commonly used physicochemical analysis methods based on apple component testing are time-consuming, expensive to run, and complex in terms of sample preparation [7,8]. Subsequently, with industrial development and technological advances, a number of rapid and non-destructive techniques for differentiating apple varieties have emerged, such as electronic noses, visible and near-infrared spectroscopy, and image processing-based methods [9,10].

Deep learning has been successfully applied as a non-destructive technique for the automatic identification, classification, and detection of fruits and vegetables with the advantages of speed, convenience, low cost, and high accuracy [11]. In particular, Convolutional Neural Networks (CNNs), deep learning-based frameworks with strong capabilities in the automatic feature learning of images, have achieved impressive results in various food and agricultural challenges [12,13]. Recently, CNNs have been used for apple recognition tasks, but mainly for quality assessment and bruise detection. For instance, Unay [14] proposed a CNN-based model to realize the quality grading of bi-colored apples using multispectral images, Lu et al. [15] applied a CNN-based model to detect immature or mature apples on trees in an orchard, Fan et al. [16] used a CNN-based model to detect defective apples on a fruit sorting machine, and Hu et al. [17] applied a CNN-based model to identify bruised apples in apple grading systems. In addition, a few studies have reported on the use of CNNs in apple variety recognition. For example, Chu et al. [18] developed a suppression mask R-CNN to recognize two varieties of apples with distinct yellow and red colors in orchard environments; Xue et al. [19] combined CNNs with a convolution autoencoder to classify 26 different fruits, including nine classes of apples; and Chen et al. [20] used a CNN-based approach to classify apple varieties by using 30 kinds of leaf images from different growth periods in nature. In general, apple quality detection and grading tasks involve fewer classes (e.g., organic/conventional apple, premium/middle/poor grades) [21,22], while the task related to apple variety classification is recommended to be performed with as many classes as possible to approximate real-world scenarios. Moreover, the apple variety classification task is challenging due to the inter-class similarities and intra-class irregularities in apple size, shape, and color [23]. Compared to binary classification, the more classes in a multiclass classification, the more difficult the task becomes, as the classification accuracy may decrease significantly as the number of classes increases [24].

Furthermore, the related work mentioned above and most applications of CNN approaches in the food field are generally focused on the performance of different models, such as the comparison of model evaluation metrics (e.g., accuracy, precision, recall) to obtain the optimal model, while fewer studies have involved and investigated the interpretability of models. However, it is important to discuss model interpretability because a deep learning model is a “black box” and its inner working mechanism is not known, and even if the model achieves good performance in applications, it is still limited by its “black box” problem and leads to “not being trusted”. Thus, the interpretability of a deep learning model is highly correlated with its credibility, which has made interpretability analysis a hot research topic in the application of deep learning in other domains [25]. In the process of human interaction with CNN models, visualization tools can give a certain level of interpretability to the model, helping people to understand its working mechanism and increasing confidence and trust in the model.

Based on the above, an automated system for apple variety classification would benefit the development of smart farming in orchards, sorting systems in the agricultural industry, and checkout systems in supermarkets [6]. In this study, we employed two frameworks of CNNs (series networks and directed acyclic graph networks) with transfer learning to automatically classify 13 types of apples. The aim of this study was not only to develop an automated system for classifying apple varieties but also to find out the impact of different CNN frameworks, network depths (under the same framework), and dataset configurations on the results of a multi-class classification task, as well as to explore the interpretability of models using visualization methods to further improve the performance and credibility of the models. The obtained results contribute to autonomous robotic fruit harvesting and post-harvest technology and further accelerate the development of agro-based industries.

Accordingly, the contributions of this study are as follows.

1. We used five CNNs from two different frameworks, i.e., series networks (AlexNet and VGG-19) and DAG networks (ResNet-18, ResNet-50, and ResNet-101), to classify 13 classes of apple. The performance of the different models was evaluated and compared in detail, and the strengths and weaknesses of each model were clarified and summarized.
2. We set up two datasets to investigate the dataset configuration on the classification results of CNN-based models. Specifically, one is a common dataset configuration, i.e., more training data–less testing data, and the other is designed to approach the reality that the testing set is infinite, i.e., less training data–more testing data.
3. We used three visualization methods (feature visualization, strongest activations, and local interpretable model-agnostic explanation techniques) step by step to reveal how the “black box” models make classification decisions.

## 2. Material and Methods

### 2.1. Fruits-360 Dataset

**Figure 1:**
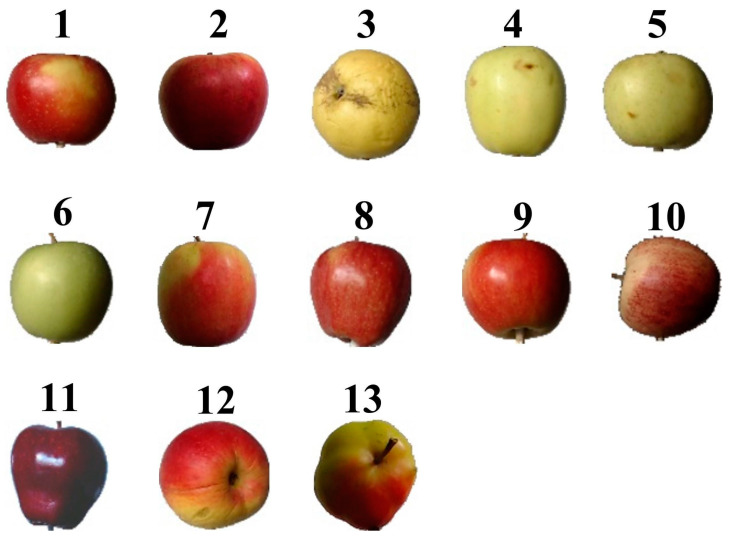
Thirteen classes of apples: 1. Braeburn, 2. Crimson Snow, 3. Golden 1, 4. Golden 2, 5. Golden 3, 6. Granny Smith, 7. Pink Lady, 8. Red 1, 9. Red 2, 10. Red 3, 11. Red Delicious, 12. Red Yellow 1, and 13. Red Yellow 2.

“Fruits-360” (https://www.kaggle.com/moltean/fruits, Version: 2020.05.18.0, accessed on 10 October 2022) is a publicly available benchmark fruit dataset [4,26], which has been used by several studies to validate their proposed models. For example, Siddiqi [27] used this dataset to classify different categories of fruits and illustrated that the Fruits-360 dataset is larger compared to other fruit datasets. Kodors et al. [28] used this dataset to classify apples and pears in order to compare the performance of different deep learning architectures. Based on this, Fruits-360 was used in this study to objectively evaluate and compare the performance of the proposed models. Fruits-360 contains 90,483 images from 131 categories of fruits and vegetables, including a total of 8538 images of 13 classes of apples from a wide range of varieties, such as Braeburn, Crimson Snow, Golden, Granny Smith, Pink Lady, and Red Delicious. Each image (100 × 100 pixels) is of a single apple on a white background, as shown in Figure 1.

### 2.2. Training and Testing Datasets Set-Up

Thirteen classes of apple images from Fruits-360 were used to build two datasets, as shown in Table 1. Dataset A was configured from the original training and testing sets provided by Fruits-360, and the validation set was a random selection of 1/5 of the images from the training set. Dataset B was an inverted version of dataset A, i.e., the testing set of dataset B corresponded to the sum of the training and validation sets of dataset A, and the sum of the training and validation sets of dataset B corresponded to the testing set of dataset A. As a result, the training-to-testing ratios for dataset A and dataset B were 2.4:1.0 and 1.0:3.7, respectively.

### 2.3. Network Architectures

Five CNNs from two different frameworks were used to investigate the effects of different structures and depths of the network on the classification results.

#### 2.3.1. Series Networks

A series network is a neural network for deep learning that has layers arranged one after the other, with only one input layer and one output layer [29]. AlexNet and VGG-19 are representative series networks that have achieved good performance in image recognition and classification. They have similar architecture but a different number of layers (depth). Specifically, AlexNet has 25 layers, including 5 convolutional layers and 3 fully connected layers, while VGG-19 has 47 layers, including 16 convolutional layers and 3 fully connected layers. More information about these two networks has been described in previous studies [30,31].

#### 2.3.2. Directed Acyclic Graph Networks

A directed acyclic graph (DAG) network is another structure of neural network used for deep learning, with layers arranged as a directed acyclic graph [29]. The residual network (ResNet) is a type of DAG network with residual (shortcut) connections that bypass the main network layers. The invention of ResNet was an important milestone in the development of CNNs [32,33]. In recent years, ResNet has outperformed previous models in image recognition and object detection [34]. There are several variants of ResNet that differ only in the number of layers (depth), so to find out the effect of network depth on the results of a classification task, we used ResNet-18, ResNet-50, and ResNet-101 in this study, and more information about ResNet has been well described in previous studies [33,35].

### 2.4. Transfer Learning

In general, CNNs are trained on large datasets of more than one million images (e.g., ImageNet) and perform best when they have deeper and more highly interconnected layers [12]. However, the currently used CNNs involving fruit and vegetable classification tasks are trained on a limited number of classes and small datasets, which can easily lead to the overfitting of deep networks, making the results unscientific and unconvincing [23]. Moreover, training these large CNNs can lead to significant drawbacks such as increased computational costs and slow-running processes [36].

To overcome the above problems, transfer learning was used. Transfer learning is a deep learning method that uses the information collected from an established model to start over with a different problem [37]. Take AlexNet as an example, as shown in Figure S1 in the Supplementary Materials. With transfer learning, the entire structure of AlexNet is divided into two parts: the pre-trained part and the replaced part. The replaced part is only a small part of the whole network, thus a small training dataset is sufficient for transfer learning. Meanwhile, transfer learning can help reduce the dependence of deep networks on computer hardware and training time [38], and more details can be found in our previous studies [31,39]. In addition, AlexNet, VGG-19, ResNet-18, ResNet-50, and ResNet-101, below, all refer to the corresponding models that were pre-trained on Imagenet and re-trained in apple recognition tasks by transfer learning.

### 2.5. Image Processing

All the images were resized to fit the input size requirements of each pre-trained network. Specifically, 227 × 227 pixels for AlexNet and 224 × 224 pixels for VGG-19, ResNet-18, ResNet-50, and ResNet-101.

### 2.6. Metrics for Performance Evaluation of CNN-Based Models

A confusion matrix is a specific table layout used to describe and visualize the performance of a trained model on a testing set [40]. Common metrics including accuracy, precision, recall, and F1-score can be computed from the confusion matrix and are used here to further reflect and compare the CNN-based models’ performance. The equations of common metrics are shown below, where TP is true positive, FP is false positive, TN is true negative, and FN is false negative.

(1) Accuracy=(TP+TN)/(TP+FP+TN+FN)

(2) Precision=TP/(TP+FP)

(3) Recall=TP/(TP+FN)

(4) F1-score=(2×Precision×Recall )/(Precision+Recall)

Accuracy refers to the overall classification accuracy, which is a sum of the total correct positive cases and the total negative cases over the total number of cases; precision is the proportion of positive cases correctly predicted by the model to the true positive cases; recall is the proportion of positive cases correctly predicted by the model to the number of positive cases predicted by the model; and F1-score is the summed average of precision and recall [41]. In addition, since our study involves a multiclassification task, the macro-average values of precision, recall, and F1-score were also calculated, i.e., macro-precision, macro-recall, and macro-F1, and the expressions are given by the following equations [20]:

(5) $$\;macro-Precision=\frac{1}{n}\sum_{i=1}^{n}Precision_{i}$$

(6) $$macro-Recall=\frac{1}{n}\sum_{i=1}^{n}Recall_{i}$$

(7) $$macro-F1=\frac{2\times macro-Precision\times macro-Recall\;}{macro-Precision+macro-Recall}$$

### 2.7. Visualization Methods

Three visualization methods were applied to deconstruct the five CNN-based models to improve their interpretability and credibility for food science applications.

#### 2.7.1. Feature Visualization

Images of the feature visualization of the last fully connected layer of each trained model were generated using the deepDreamImage technique [31].

#### 2.7.2. Strongest Activations

One image of each type of apple was randomly selected from the testing set and fed into the trained model to show the strongest activations of the last convolutional layer using the method “Visualize Activations of a Convolutional Neural Network” in Mathworks.

#### 2.7.3. Local Interpretable Model-Agnostic Explanations

One image of an apple was randomly selected from the testing set and fed into the trained model to show the corresponding local interpretable model-agnostic explanations (LIME) image using the method “local interpretable model-agnostic explanations” in Mathworks.

### 2.8. Computer Configuration and Model Hyperparameters

To compare the performance of different CNN-based models, all models were implemented using MATLAB R2020b version, running on the same personal desktop with an Intel(R) Core i7-9700k CPU*1 and NVIDIA^®^ GeForce RTX 2060 SUPER GPU*1, and trained by Adaptive Moment Estimation (ADAM). In addition, the same model hyperparameters were adopted: initial learning rate = 0.00001, learn rate drop factor = 0.1, learn rate drop period= 10, minibatch size = 64, and Max Epochs = 15. The training progress of the five models on dataset A and dataset B is shown in Figure S2 and Figure S3 in the supplementary file, respectively, including classification accuracy and cross-entropy loss for each epoch of training and validation [42].

## 3. Results and Discussion

### 3.1. Performance of the Different Trained Models

**Figure 2:**
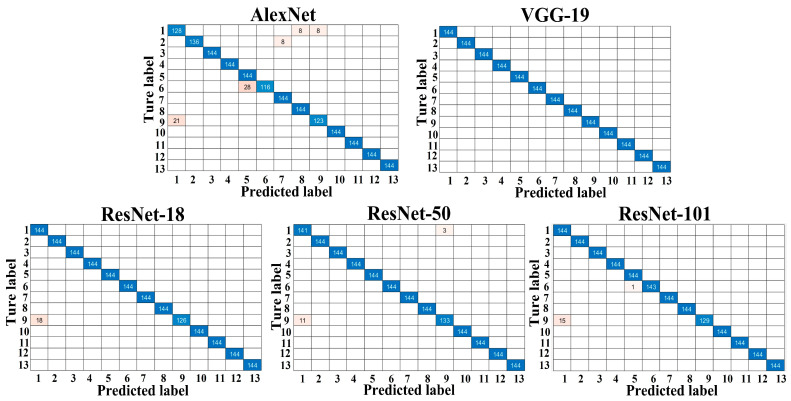
Confusion matrix of the different trained models for dataset A.

**Figure 3:**
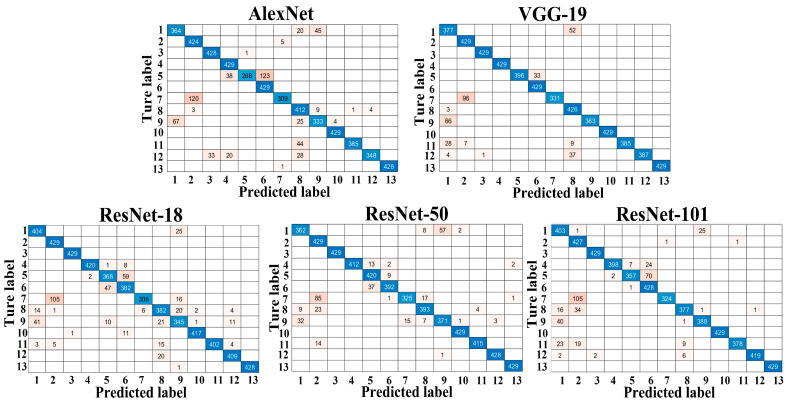
Confusion matrix of the different trained models for dataset B.

The confusion matrices for the testing sets of dataset A and dataset B are shown in Figure 2 and Figure 3, respectively, with the correct predictions for each category located on the diagonal of the table and marked in blue and the incorrect predictions for each category marked in pink. Compared to Figure 2, the number of misclassified images for each trained model in Figure 3 increased noticeably, resulting in a decrease in the overall classification accuracy of each trained model. As shown in Table 2, the overall classification accuracy achieved by all models on dataset A was significantly higher than that of dataset B, indicating that the dataset configuration had a significant effect on the classification results. Specifically, models trained with a relatively large training set and tested on a smaller testing set achieved high overall classification accuracies, especially for VGG-19 and the three ResNets, which achieved over 99% overall classification accuracy on dataset A. Moreover, in a real-world scenario, testing sets tend to be infinite or unlimited, so to approach reality [43], we set up dataset B (the size of the testing set is 3.7 times greater than the training set) for further comparison of the performance of the five models. As expected, the reduction in the size of the training set and the increase in the size of the testing set posed a challenge to the models, resulting in a significant decrease in the overall classification accuracy of all models, as well as other metrics in Table 3 and Table 4. This phenomenon is consistent with our previous research, such as the classification of tomato varieties using CNN-based models [44]. However, VGG-19 and the three ResNets still maintained good performance on dataset B, with an overall classification accuracy greater than 91.9% and a macro-F1 greater than 92.1%.

Compared to other studies [19,20,45], which generally employed only one dataset (more training data–less testing data) to evaluate the performance of CNN-based models, we set up these two datasets in order to find out the effect of different sizes of training sets on apple variety classification results, such as classification accuracy, training time, etc. This is because it is often difficult to obtain large training sets in practice, and even when large training sets are available, manually labeling them is time-consuming and laborious work [39]. In addition, a larger training set means more computer resource consumption and longer training time for the same model, as shown in Table 2. For this reason, it would be beneficial for the practical use of CNNs if they could produce desirable and reliable results based on a relatively small training set. Furthermore, it was also found that for the same type of network (series or DAG network), the model size, overall classification accuracy, and training and testing times increased as the model depth (number of layers) increased. This phenomenon is consistent with many recent studies that suggest that network depth is a key factor in leading the results in image classification tasks because CNNs automatically learn and integrate (low/med/high-level) features from the training set to classify images, and the level of features can be improved as the network depth increases, which contributes to an improvement in classification accuracy to some extent [33].

In real-world applications, the ultimate goal of a multi-class classification task is to achieve the most accurate recognition of a single image in the shortest possible time [43]. The recognition time for a single image is a key point to consider when evaluating the performance of different models, as it is directly related to the efficiency of the model in practice, whereas model training is a one-off activity, or at most periodic to maintain and update its performance [44]. Therefore, although the training times for the models were long in this case, ranging from a few minutes to several hours, they were still acceptable and would be further reduced with improvements in computer hardware [39]. For the series networks, VGG-19 achieved the highest accuracy (i.e., 100% for dataset A and 93.9% for dataset B) and macro-F1 (i.e., 100% for dataset A and 94.4% for dataset B) on both datasets. For the DAG networks, ResNet-50 achieved the highest accuracy (i.e., 99.3% for dataset A and 93.9% for dataset B) and macro-F1 (i.e., 99.3% for dataset A and 94.1% for dataset B) on both datasets. When comparing these two models, we can easily conclude that VGG-19 has the highest accuracy among the five models. However, the average time for VGG-19 to recognize an image was approximately 4.7 times longer than that of ResNet-50, which reduces its efficiency in practice. Therefore, ResNet-50 is more practical than VGG-19. Based on the above, the strengths and weaknesses of each model are summarized in Table 5, which can be used as a guide for the selection of a suitable model in practice. In addition, as all models achieved excellent performance on dataset A, a comparison of the feature visualization, strongest activation, and LIME of the different models trained on dataset A is presented in the following sections.

### 3.2. Model Interpretability Analysis

#### 3.2.1. Feature Visualization

**Figure 4:**
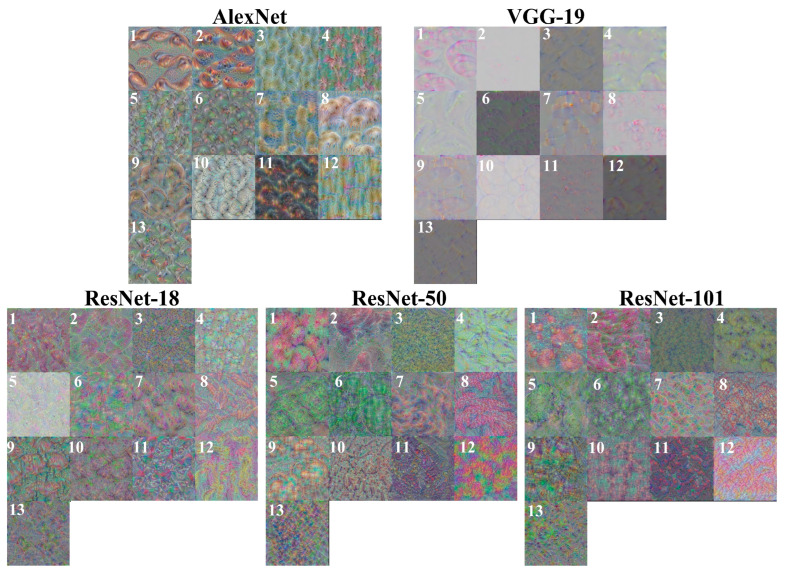
Feature visualization of the last fully connected layer of the different trained models, and the numbers on the images correspond to the class of apples.

Features are generally the physical characteristics of an object that can be used to distinguish it from other objects. A fruit has many physical characteristics, including color, texture, shape, and size, which are used by traditional fixed-feature-based machine learning methods for recognition and classification tasks, such as detecting the defects or maturity of fruits [46,47]. However, such fixed, simple, feature-based classifiers are not robust or suitable for complex tasks because fruits have many inter-class and intra-class similarities and variations, and especially inter-class similarities and intra-class variations pose significant challenges [6,23]. In contrast to simple fixed-feature-based machine learning methods, CNNs are able to automatically learn and integrate features from training images and use them for classification tasks [44]. Specifically, the convolutional layers act as feature extractors for the input images, whose dimensionality is subsequently reduced by the pooling layers, and the fully connected layers act as classifiers [48]. Therefore, in this study, the feature visualization of the last fully connected layer of the different trained models was used to explain to us how the trained CNNs build an understanding of apple images, i.e., the common and high-level features of each type of apple learned by the trained models from the training set, as shown in Figure 4 [49].

It is clear that the feature visualization images of different trained models differ in pattern or style even for the same class of apples, suggesting that different models interpret the same class of apple in different ways. At the same time, although some classes of apples are very similar in appearance, their corresponding feature visualization images generated by different models were still different, suggesting that different CNN-based models learned the true differences between classes of apples [44]. For instance, Golden 3 and Granny Smith (classes 5 and 6 in Figure 1) had distinctly different feature visualization images for each model despite their similar colors, shapes, and sizes. Moreover, for series networks, the feature visualization images generated by AlexNet for each type of apple show many repeat patterns, while the feature visualization images generated by VGG-19 show a lot of random stripes, which are more abstract than those generated by AlexNet. For DAG networks, the feature visualization images generated by the three ResNet-based models were also different and abstract, which is difficult to interpret. This phenomenon is due to the different depths of the models because CNNs typically build an understanding of images in a hierarchical way over many layers, where earlier layers learn basic and low-level features such as colors, edges, textures, or shapes, and later layers learn and integrate simple features (learned by earlier layers) into increasingly complex and abstract features, such as patterns, parts, or objects, so that the last fully connected layer learns the high-level features of each class and uses them for prediction, but sometimes the high-level features are too abstract to be interpreted [50,51]. Based on this, since deeper layers can learn the combinations of features learned by the previous layers, the deeper ResNet-based model implies more convolutional layers, which can extract more advanced and complex features than the relatively shallow model [29].

#### 3.2.2. Strongest Activations

**Figure 5:**
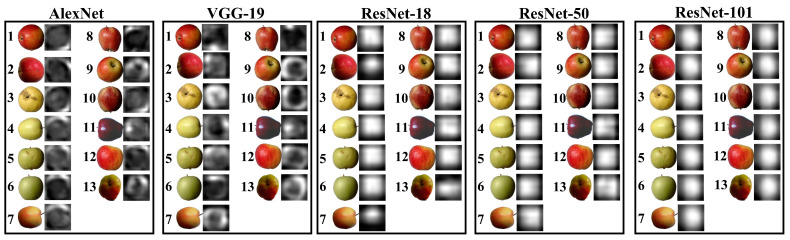
Apple images of each class and the corresponding strongest activations of the last convolutional layer of the different trained models. The numbers on the images correspond to the class of apple.

The purpose of presenting the strongest activations is to observe and compare how trained models recognize apples. Figure 5 shows one randomly selected image from each type of apple in the testing set and the corresponding strongest activations generated by the last convolutional layer of the different trained models. In the strongest activation images, strong positive activation is shown by white pixels and strong negative activation is shown by black pixels [31]. We focus on the white areas in the images, as they indicate the areas recognized by the trained models [52]. Interestingly, for series networks, white areas show that AlexNet and VGG-19 classify apples based on their contours or shapes, whereas for DAG networks, ResNet-based models classify apples based on their entire region. These findings are exclusive because, to the best of our knowledge, there are no related papers that reveal how a CNN-based model recognizes apples, and these results also suggest that models with different frameworks recognize apples in different ways.

#### 3.2.3. Local Interpretable Model-Agnostic Explanations

Since LIME typically uses simple and more interpretable models (e.g., linear models or decision tree models) to locally approximate the predictions of the target black-box model, LIME was applied here to figure out how CNN-based models make classification decisions on apple types in order to further improve the interpretability of the models [50,53].

**Figure 6:**
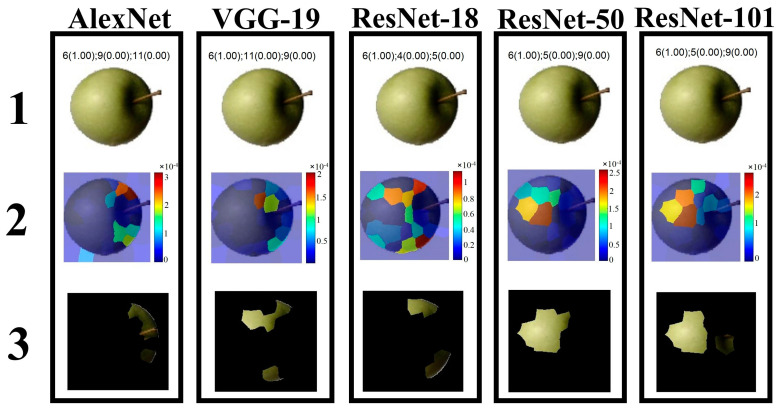
Understanding CNN-based models by using the LIME technique. The number on the image represents the corresponding row.

Figure 6 shows the feature importance maps corresponding to each model as determined by the LIME technique. Specifically, the first row shows the classification results of the same apple image by the different models, i.e., the three categories that received the highest classification probabilities are displayed at the top of the image. The second row shows the recognition region of the image that the model used to classify. The third row shows the most important features determined by each model [29]. For instance, in row 1 column 1, AlexNet classified the apple image as Class 6 (Granny Smith) with 100% probability, and Class 9 (Red 2) and Class 11 (Red Delicious) with 0 probability. For row 2 column 1, the feature map shows which regions of the image were important for the classification of the apple (Class 6). According to the chromaticity bar, the red regions have a high importance, i.e., AlexNet focuses on the upper right region of the apple to predict Class 6, and the prediction accuracy decreases when these regions are removed [53]. For row 3 column 1, it is a masked image and the visible regions need to be focused on as it indicates the most important features identified by AlexNet, corresponding to the important regions in the row 2 column 1 image. On this basis, we can find that different models identify the important features in different regions.

Based on the above, Section 3.2 provides insight into the five CNN-based models through three visualization methods to explore the models’ working mechanisms in this task. Specifically, feature visualization images show the different understanding of apple images by different trained models, while the strongest activations and LIME images show how and why different trained models make classification decisions. Unfortunately, some behaviors of the different trained models mentioned in this section, such as why series networks classify apples based on their contours or shapes while DAG networks classify apples based on their entire region, or why different models identify the important features of apples in different regions, are currently unexplained, as deep learning is still a “black box” technology and its internal mechanisms are not yet fully understood [25,54]. However, gaining insight into the internal workings of CNNs is valuable, as by revealing the contribution (or not) of various features to models’ predictions, we can compare differences in the evidence used by models and professionals in identifying and classifying apples. These results help us to explain model predictions and build trust in deep learning for practical applications [50].

## 4. Conclusions

Highly automated recognition and classification systems for fruit varieties are very important and necessary in the future of agriculture and food because they can significantly reduce labor costs and improve the economic efficiency of fruits from harvest to market. Five CNNs from two different structures, i.e., series networks (AlexNet and VGG-19) and DAG networks (ResNet-18, ResNet-50, and ResNet-101), were used on two datasets to identify and classify 13 classes of apples. Important findings were that (1) the dataset configuration had a significant effect on the classification results, as the overall classification accuracy of all models exceeded 96.1% on dataset A (training-to-testing = 2.4:1.0) compared to 89.4–93.9% on dataset B (training-to-testing = 1.0:3.7); (2) for the same framework of a network (series or DAG network), the model sizes, accuracies, and training and testing times increased as the model depth (number of layers) increased; and (3) feature visualization found that the understanding (learned features) of the apples was different for different models, and strongest activations images revealed that series networks classified apples based on their contours or shapes while DAG networks classified apples based on their entire region, and the LIME technique showed the important features used by the models to make classification decisions. The obtained results not only show the applicability of CNNs in apple recognition tasks but also contribute to the interpretability exploration of CNN-based models and further provide guidance for future applications of deep learning-based methods in agriculture.

Our future work is mainly based on the following aspects: (1) Increasing the number of apple classes used for classification, as the number of apple classes used in this study is insufficient compared to the global market. (2) Developing a new method that can automatically perform hyperparameter optimization of the model, as CNN-based methods are sensitive to hyperparameter optimization, which is usually an empirically based and time-consuming task. (3) We will continue to investigate the interpretability of CNN-based models to reveal their inner workings and mechanisms in order to improve the credibility and trust of users in the food field.

## Figures and Tables

**Table 1:** The number of images per class for training, validation, and testing in the two datasets.

Class	Dataset A			Dataset B		
	Training Set	Validation Set	Testing Set	Training Set	Validation Set	Testing Set
1 Braeburn	344	85	144	116	28	429
2 Crimson Snow	344	85	144	119	28	429
3 Golden 1	344	85	144	116	28	429
4 Golden 2	344	85	144	116	28	429
5 Golden 3	344	85	144	116	28	429
6 Granny Smith	344	85	144	116	28	429
7 Pink Lady	344	85	144	116	28	429
8 Red 1	344	85	144	116	28	429
9 Red 2	344	85	144	116	28	429
10 Red 3	344	85	144	116	28	429
11 Red Delicious	344	85	144	116	28	429
12 Red Yellow 1	344	85	144	116	28	429
13 Red Yellow 2	344	85	144	116	28	429
Total	4472	1105	1872	1508	364	5577

**Table 2:** Comparison of the different trained models.

	AlexNet	VGG-19	ResNet-18	ResNet-50	ResNet-101
Type of network	Series	Series	DAG	DAG	DAG
Connections	none	none	78 × 2 table	192 × 2 table	379 × 2 table
Running hardware	GPU	GPU	GPU	GPU	CPU
Layers	25	47	71	177	347
Model size (MB)	204	495	40	84	151
Dataset A (training-to-testing = 2.4:1.0)					
Training time (s)	85.4	5716.6	231.3	952.0	20548.0
Testing time (s)	1.0	22.0	1.9	4.6	62.7
Classification time for one image (ms)	0.5	11.8	1.0	2.5	33.5
Overall classification accuracy (%)	96.1	100.0	99.0	99.3	99.2
Dataset B (training-to-testing = 1.0:3.7)					
Training time (s)	29.0	1707.6	74.6	295.6	6971.6
Testing time (s)	2.3	54.3	4.7	11.8	188.2
Classification time for one image (ms)	0.4	9.7	0.8	2.1	33.7
Overall classification accuracy (%)	89.4	93.9	91.9	93.9	93.0

**Table 3:** Precision, recall, and F1-score of AlexNet- and VGG-19-based models on dataset A and dataset B.

Dataset A		AlexNet			VGG-19	
Class	Precision (%)	Recall (%)	F1-Score (%)	Precision (%)	Recall (%)	F1-Score (%)
1	85.9	88.9	87.4	100.0	100.0	100.0
2	100.0	94.4	97.1	100.0	100.0	100.0
3	100.0	100.0	100.0	100.0	100.0	100.0
4	100.0	100.0	100.0	100.0	100.0	100.0
5	83.7	100.0	91.1	100.0	100.0	100.0
6	100.0	80.6	89.3	100.0	100.0	100.0
7	94.7	100.0	97.3	100.0	100.0	100.0
8	94.7	100.0	97.3	100.0	100.0	100.0
9	93.9	85.4	89.4	100.0	100.0	100.0
10	100.0	100.0	100.0	100.0	100.0	100.0
11	100.0	100.0	100.0	100.0	100.0	100.0
12	100.0	100.0	100.0	100.0	100.0	100.0
13	100.0	100.0	100.0	100.0	100.0	100.0
macro-	96.4	96.1	96.2	100.0	100.0	100.0
Dataset B						
Class						
1	84.5	84.8	84.6	78.9	87.9	83.2
2	77.5	98.8	86.9	80.3	100.0	89.1
3	92.8	99.8	96.2	99.8	100.0	99.9
4	88.1	100.0	93.7	100.0	100.0	100.0
5	99.6	62.5	76.8	100.0	92.3	96.0
6	77.7	100.0	87.5	92.9	100.0	96.3
7	98.1	72.0	83.0	100.0	77.2	87.1
8	77.9	96.0	86.0	81.3	99.3	89.4
9	86.0	77.6	81.6	100.0	84.6	91.7
10	99.1	100.0	99.5	100.0	100.0	100.0
11	99.7	89.7	94.4	100.0	89.7	94.6
12	98.9	81.1	89.1	100.0	90.2	94.8
13	100.0	99.8	99.9	100.0	100.0	100.0
macro-	90.8	89.4	90.1	94.9	93.9	94.4

**Table 4:** Precision, recall, and F1-score of the three ResNet-based models on dataset A and dataset B.

Dataset A		ResNet-18			ResNet-50			ResNet-101	
Class	Precision (%)	Recall(%)	F1-Score (%)	Precision (%)	Recall(%)	F1-Score (%)	Precision (%)	Recall(%)	F1-Score (%)
1	88.9	100.0	94.1	92.8	97.9	95.3	90.6	100.0	95.1
2	100.0	100.0	100.0	100.0	100.0	100.0	100.0	100.0	100.0
3	100.0	100.0	100.0	100.0	100.0	100.0	100.0	100.0	100.0
4	100.0	100.0	100.0	100.0	100.0	100.0	100.0	100.0	100.0
5	100.0	100.0	100.0	100.0	100.0	100.0	99.3	100.0	99.6
6	100.0	100.0	100.0	100.0	100.0	100.0	100.0	99.3	99.6
7	100.0	100.0	100.0	100.0	100.0	100.0	100.0	100.0	100.0
8	100.0	100.0	100.0	100.0	100.0	100.0	100.0	100.0	100.0
9	100.0	87.5	93.3	97.8	92.4	95.0	100.0	89.6	94.5
10	100.0	100.0	100.0	100.0	100.0	100.0	100.0	100.0	100.0
11	100.0	100.0	100.0	100.0	100.0	100.0	100.0	100.0	100.0
12	100.0	100.0	100.0	100.0	100.0	100.0	100.0	100.0	100.0
13	100.0	100.0	100.0	100.0	100.0	100.0	100.0	100.0	100.0
macro-	99.1	99.0	99.1	99.3	99.3	99.3	99.2	99.1	99.2
Dataset B									
Class									
1	87.4	94.2	90.7	89.8	84.4	87.0	83.3	93.9	88.3
2	79.4	100.0	88.5	77.9	100.0	87.6	72.9	99.5	84.1
3	99.8	100.0	99.9	100.0	100.0	100.0	99.5	100.0	99.7
4	99.5	97.9	98.7	100.0	96.0	98.0	99.5	92.8	96.0
5	86.4	85.8	86.1	89.4	97.9	93.5	97.8	83.2	89.9
6	83.0	89.0	85.9	97.0	91.4	94.1	82.0	99.8	90.0
7	98.1	71.8	82.9	95.6	75.8	84.6	99.7	75.5	85.9
8	87.2	89.0	88.1	92.5	91.6	92.0	95.9	87.9	91.7
9	84.8	80.4	82.5	86.5	86.5	86.5	93.7	90.4	92.0
10	99.3	97.2	98.2	99.3	100.0	99.6	100.0	100.0	100.0
11	100.0	93.7	96.7	99.0	96.7	97.8	99.7	88.1	93.5
12	95.6	95.3	95.4	99.3	99.8	99.5	99.8	97.7	98.7
13	100.0	99.8	99.9	99.3	100.0	99.6	100.0	100.0	100.0
macro-	92.3	91.9	92.1	94.3	93.9	94.1	94.1	93.0	93.6

**Table 5:** Strengths and weaknesses of each trained model.

	Strengths	Weakness
AlexNet	Fast training and classifying; good practicality	Low accuracy
VGG-19	High accuracy	Slow training and classifying
ResNet-18	Fast training and classifying; high accuracy and good practicality	
ResNet-50	High accuracy, good practicality	
ResNet-101	High accuracy	Dependence on high-performance computers; slow training and classifying

## Data Availability

The datasets generated during the current study are available from Fruits-360 (https://www.kaggle.com/moltean/fruits, Version: 2020.05.18.0, accessed on 10 October 2022).

## Supplementary Materials

The following supporting information can be downloaded at: https://www.mdpi.com/article/10.3390/foods12040885/s1, Figure S1—Schematic of a pre-trained network with transfer learning, taking AlexNet as an example; Figure S2—Classification accuracy and cross-entropy loss for each epoch of training and validation on dataset A; Figure S3—Classification accuracy and cross-entropy loss for each epoch of training and validation on dataset B.
