# Supplementary material for: Deep Learning-Based Intelligent Apple Variety Classification System and Model Interpretability Analysis
Source: Foods. 2023 Feb 19;12(4):885. doi: 10.3390/foods12040885 (PMC9956933; doi:10.3390/foods12040885)

Figure.S1 Schematic of a pre-trained network with transfer learning, taking AlexNet as an example.

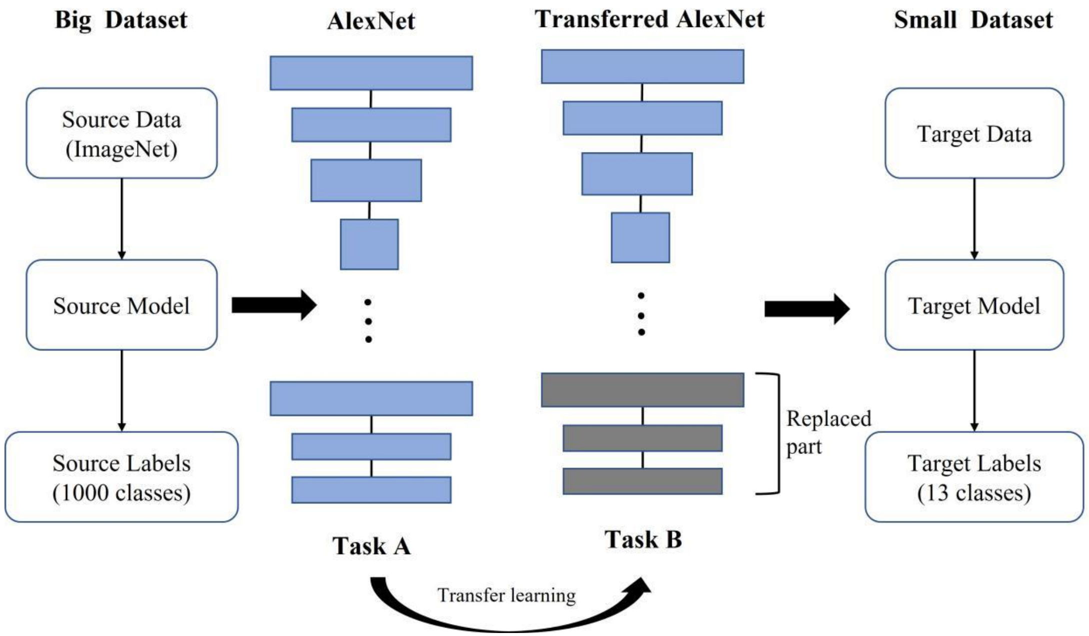

Figure.S2 Classification accuracy and cross-entropy loss for each epoch of training and validation on dataset A.

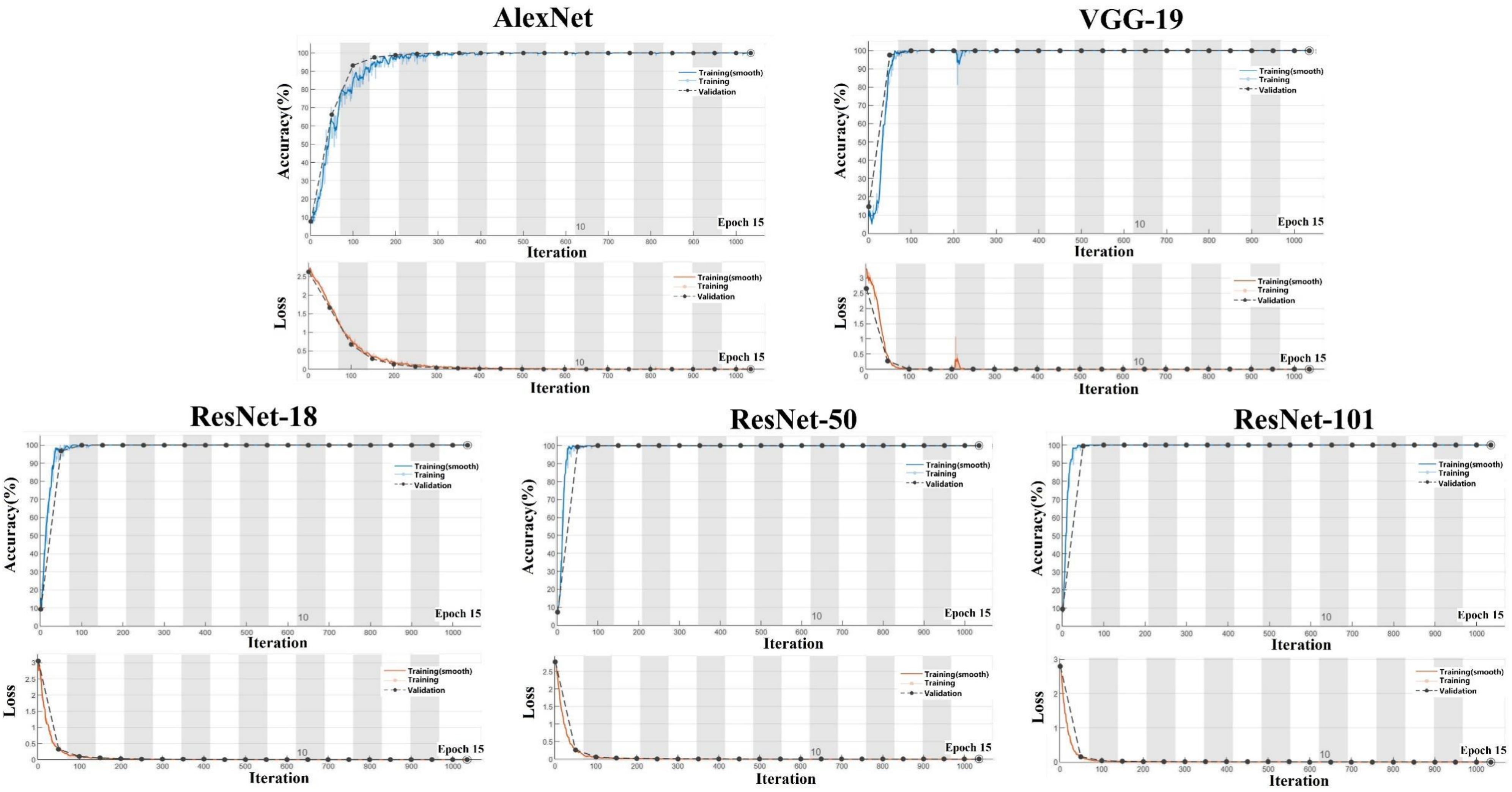

Figure.S3 Classification accuracy and cross-entropy loss for each epoch of training and validation on dataset B.

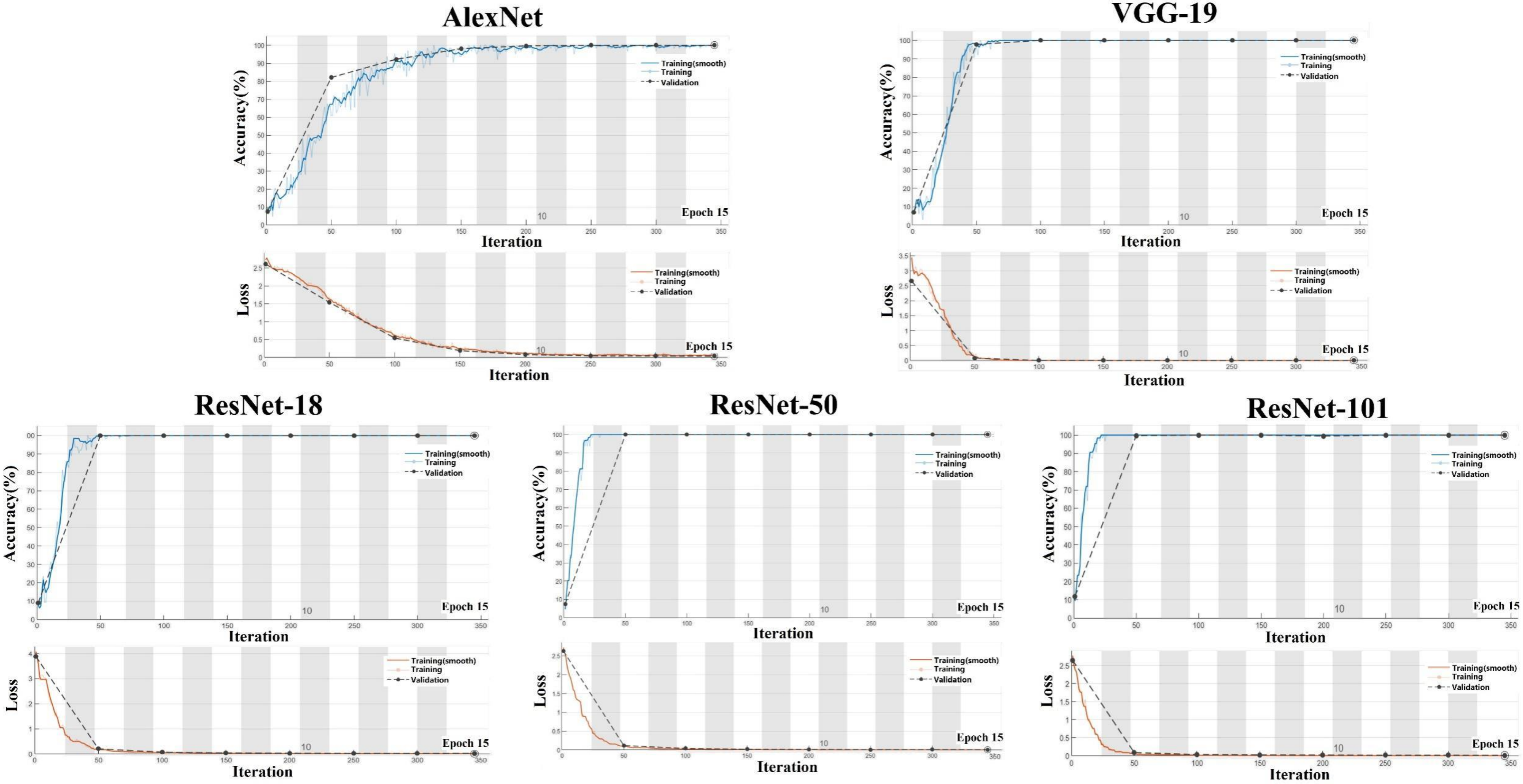

Supplement: Supplementary file 1 [file foods-12-00885-s001.zip › foods-2141435-supplementary.pdf]
